# Concepts and conjectures concerning predatory performance of myxobacteria

**DOI:** 10.3389/fmicb.2022.1031346

**Published:** 2022-09-29

**Authors:** Kayleigh E. Phillips, Shukria Akbar, D. Cole Stevens

**Affiliations:** ^1^Department of BioMolecular Sciences, The University of Mississippi, Oxford, MS, United States; ^2^Division of Pharmaceutical Sciences, School of Pharmacy, University of Wisconsin-Madison, Madison, WI, United States; ^3^Department of Bacteriology, University of Wisconsin-Madison, Madison, WI, United States

**Keywords:** myxobacteria, predation, specialized metabolism, predator–prey, microbial community structure

## Abstract

Myxobacteria are excellent model organisms for investigation of predator–prey interactions and predatory shaping of microbial communities. This review covers interdisciplinary topics related to myxobacterial predation and provides current concepts and challenges for determining predatory performance. Discussed topics include the role of specialized metabolites during predation, genetic determinants for predatory performance, challenges associated with methodological differences, discrepancies between sequenced and environmental myxobacteria, and factors that influence predation.

## Introduction

Myxobacteria are generalist predators that help recycle soil nutrients and shape microbial community structure (Johnke et al., 2014; Li et al., 2017; Nair and Velicer, 2021; Petters et al., 2021). Unlike obligate predatory bacteria, like *Bdellovibrio bacteriovorus*, which invade prey cells individually, myxobacteria are social predators capable of extracellular lysis of prey and subsequent swarming to claim released biomacromolecules for nutrition (Perez et al., 2016; Thiery and Kaimer, 2020). This swarming motility and other cooperative features such as fruiting body formation make myxobacteria an excellent model for investigation of multicellularity (Shimkets et al., 2006; Zusman et al., 2007; Bretl and Kirby, 2016; Munoz-Dorado et al., 2016; Schumacher and Sogaard-Andersen, 2017). During predation, myxobacteria secrete a combination of lytic enzymes and toxic, specialized (also referred to as secondary) metabolites to lyse prey microbes (Xiao et al., 2011; Thiery and Kaimer, 2020; Arend et al., 2021). A type IV filament-like Kil protein from *Myxococcus xanthus* has also been found to enable contact-dependent predation (Seef et al., 2021). Deemed “gifted” due to extraordinarily large genomes densely packed with specialized metabolite biosynthetic gene clusters (BGCs) (Baltz, 2017a,b, 2019, 2021), myxobacteria are a coveted source of lead compounds for drug discovery (Landwehr et al., 2016; Herrmann et al., 2017; Bader et al., 2020). These various attributes of myxobacteria combined with a global presence in soils and marine sediments (Dawid, 2000; Petters et al., 2021; Wang et al., 2021b) engender broad multidisciplinary interest, and predation is a unifying theme across disciplines. Antibiotic discovery associated with predatory lifestyles (Panter et al., 2018), evolution during predator–prey interactions (Nair et al., 2019), predatory nutrient cycling in soils (Petters et al., 2021), and the application of myxobacteria as biocontrol agents (Ye et al., 2020) are all examples of predation-aligned research.

This review utilizes discoveries across disciplines to discuss ongoing investigation of myxobacterial predatory performance. Few studies have thoroughly established the prey range of individual myxobacteria, and even fewer have compared predatory capacity across myxobacterial taxa. Ranking predatory performance of 17 strains of *M. xanthus* using a cohort of 12 prey bacteria, Morgan et al. reported specialized predatory performance on individual prey species (Morgan et al., 2010). The authors suggest that predatory specialization may result from predator or prey adaptations due to higher environmental encounter rates. A broader investigation of prey range, including 113 environmental isolates of myxobacteria and clinically relevant prey bacteria, revealed predatory activity to be unrelated to phylogeny (Livingstone et al., 2017). These examples suggest that predator–prey interactions incur dynamic, rapid phenotypic variation and environmental specialization of generalist predators. Herein, we provide inferences and challenges associated with determining predatory performance as well as factors known to influence myxobacterial predation.

### Conjectured association of specialized metabolism and predation

**Figure 1:**
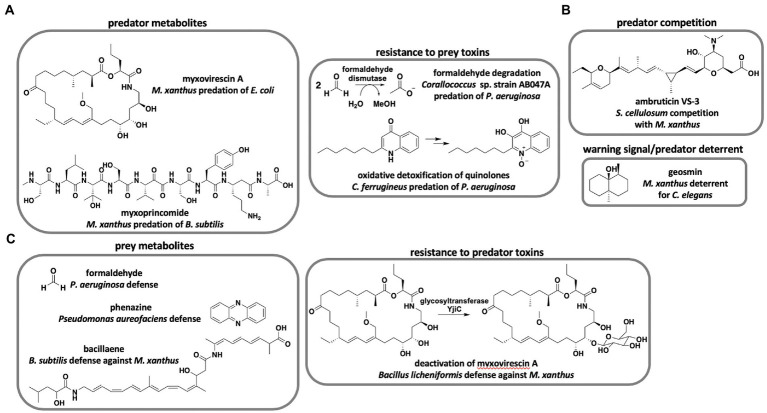
Roles of specialized metabolites in myxobacterial predator–prey interactions. **(A)** Metabolites and metabolic transformations that benefit predation of prey (Xiao et al., 2011; Muller et al., 2016; Sutton et al., 2019; Akbar et al., 2022). **(B)** Myxobacterial metabolites not directly involved in predation of prey (Marcos-Torres et al., 2020; Zaroubi et al., 2022). **(C)** Prey metabolites and metabolic transformations that inhibit myxobacterial predation (Bull et al., 2002; Muller et al., 2014; Sutton et al., 2019; Wang et al., 2019).

Although over 100 unique metabolites and 500 analogs have been reported by drug discovery efforts from myxobacteria (Herrmann et al., 2017), only two metabolites have been determined to benefit predation directly (Figure 1A). Genetic knockouts of the myxovirescin hybrid polyketide synthase (PKS)-nonribosomal peptide synthetase (NRPS) BGC resulted in mutant *M. xanthus* strains defective in killing *Escherichia coli* compared to myxovirescin-producing strains (Xiao et al., 2011). Similarly, random transposon insertion revealed that the non-ribosomal peptide myxoprincomide contributes to *M. xanthus* predation of *Bacillus subtilis* (Muller et al., 2016). Although not directly shown to impact predation, co-cultivation of epothilone-producing and non-producing *Sorangium* strains resulted in increased production of the antifungal metabolite (Li et al., 2013, 2014). Li et al. suggest increased epothilone production during co-cultivation to be a cooperative predation mechanism between *Sorangium* strains to consume fungal competitors. Despite the limited evidence that predation benefits from discovered antibiotics, predatory lifestyles are often cited as a motivating factor for continued drug discovery from myxobacteria (Korp et al., 2016; Perez et al., 2020). Currently, there is no clear correlation between antibiotic repertoire and predatory performance of myxobacteria. Pan-genome and predatory range analysis of 23 *Corallococcus* spp. revealed incongruencies between BGC content and predation (Livingstone et al., 2018b). The authors suggest that predation is partially dependent on horizontally acquired genes in the accessory pan-genome of *Corallococcus* spp. Coinciding with the previously mentioned prey range study, both predator and prey phylogeny fail to predict predatory activity (Livingstone et al., 2017). Interestingly, a metabolomic study surveying ~2,300 myxobacterial extracts observed a correlation between detected specialized metabolites and taxonomic distance (Hoffmann et al., 2018). Essentially, hierarchical clustering of metabolite profiles from axenically grown myxobacteria mapped to taxonomy with genus-level clustering and species-level clustering of *Myxococcus* spp. While the metabolic profiles and phylogeny of myxobacteria are correlated, predatory performance is not predictable from predator or prey phylogeny. These studies provide a compelling disconnect from the assumption that specialized metabolites primarily benefit predation. Axenic cultivation conditions used by Hoffman et al. to generate myxobacterial extracts conceivably limit ecological relevance. However, a transcriptomic study focused on *M. xanthus* predation of *E. coli* revealed that the predator was constitutively toxic in the presence/absence of prey and instead regulated feeding when exposed to macromolecular nutrients from pre-lysed prey (Livingstone et al., 2018a). We suggest that horizontally-acquired BGCs unique to individual strains of myxobacteria may account for species-level predatory specialization. Although, the large sizes of modular BGC-types known to benefit predation [83 kb myxovirescin BGC (Simunovic et al., 2006), 48 kb myxoprincomide BGC (Cortina et al., 2012)] possibly limit horizontal gene transfer, other predatory elements such as lytic enzymes, outer-membrane vesicles, and contact-dependent features are better suited for adaptive specialization. Further investigation of pan-genome plasticity and adaptability of secondary metabolism is required to determine if predatory specialization impacts metabolic profiles of myxobacteria. Perhaps, the role of specialized metabolites in prey killing is overstated. During co-culture conditions between the myxobacteria *Sorangium cellulosum* and *M. xanthus*, *S. cellulosum* prevents *M. xanthus* fruiting body formation (Marcos-Torres et al., 2020; Figure 1B). The antifungal metabolite ambruticin *VS*-3 produced by *S. cellulosum* inhibits *M. xanthus* fruiting body formation and interrupts induction of sporulation during starvation conditions. Marcos-Torres et al. suggest that induction of *M. xanthus* sporulation despite nutrient availability enables *S. cellulosum* to outcompete the neighboring predator. Differences in secondary metabolite profiles have been implicated in a similar inhibition of sporulation during intraspecific competition between *M. xanthus* strains (Fiegna and Velicer, 2005; Krug et al., 2008). Monitoring territoriality between *M. xanthus* and *Myxococcus virescens*, Smith and Dworkin proposed that secreted bacteriocins afford *M. virescens* a competitive advantage over *M. xanthus* (McCurdy and MacRae, 1974; Smith and Dworkin, 1994). These observations present an alternative role of specialized metabolites during predator competition for nutrients. Production of the ubiquitous, volatile terpene geosmin by *M. xanthus* during exponential growth serves as a warning signal to dissuade the bacteriophagous nematode *Caenorhabditis elegans* (Zaroubi et al., 2022; Figure 1B). The geosmin BGC is also present in genomes from nearly every sequenced myxobacteria as well as many other natural product-producing bacteria (Gregory et al., 2019). This provides an example of myxobacteria using specialized metabolite production defensively as prey to discourage predatory nematodes. Altogether, the premise that predatory myxobacteria are an excellent source of therapeutic leads holds regardless of utility, and incongruencies between predatory performance and specialized metabolism combined with limited examples of predation-influencing metabolites impede genetic determination of prey range from biosynthetic capacity.

## Absence of general genetic indicators for predatory performance

There are currently no general genetic determinants that indicate predatory performance of myxobacteria. However, genetic features linked to predation from specific predator–prey pairings have been observed. A genome-wide association study including genome data from 29 myxobacteria and predation assays for 10 prey bacteria revealed 139 “predation genes,” and formaldehyde dismutase was observed to correlate with superior predation of *Pseudomonas aeruginosa* (Sutton et al., 2019; Figure 1A). Comparative genome analysis of candidate biocontrol agent *Corallococcus* sp. strain EGB revealed that abundant extracellular chitinases, β-(1,3)-glucanases, and proteases are likely involved in the predation of phytopathogenic fungi (Zhou et al., 2019; Zhao et al., 2021). Investigation of myxobacterial response to prey quorum signals revealed that oxidative degradation of toxic alkyl quinolones produced by *P. aeruginosa* benefits myxobacterium *Cystobacter ferrugineus* predation of the opportunistic pathogen (Akbar et al., 2022; Figure 1A). Conversely, studies focused on particular predator–prey interactions have also discovered features associated with prey avoidance of myxobacteria. Prey avoidance mechanisms include biofilm formation and mucoid conversion (DePas et al., 2014; Nair et al., 2019; Akbar and Stevens, 2021), sporulation (Muller et al., 2014, 2015, 2016), secretion of toxic or inhibitory metabolites (Bull et al., 2002; Muller et al., 2014; Sutton et al., 2019; Lee et al., 2020), and resistance to toxins produced by myxobacteria (Wang et al., 2019; Figure 1C). Although comparative genomic studies identify candidate genes associated with predation, identified genes often encode basic features of generalist predators or specialized traits assumed to result from frequently encountered prey phenotypes. If rapid adaptation accounts for differences in predatory performance amongst myxobacteria, general genetic determinants that broadly indicate prey range will remain elusive.

## Established concepts challenged by differences in methodology

**Figure 2:**
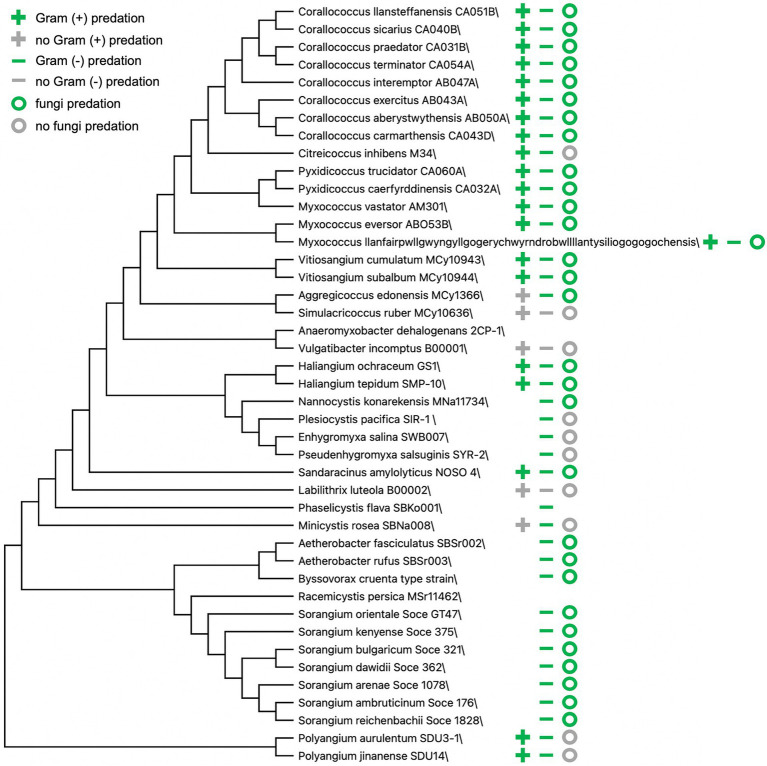
Predation data from myxobacteria type strain announcements and descriptions. Bootstrap consensus tree (300 replicates) using the Minimum Evolution method in MEGA X (Kumar et al., 2018). Absence of symbol(s) indicates no available data present in corresponding myxobacteria descriptions.

Technological and methodological advances over decades have introduced challenges to traditionally accepted theories and premises related to predatory myxobacteria. The transition from morphology-based classification of myxobacteria to a combination of traditional methods and comparative genomics has resulted in various taxonomic reassignments and proposed updates (Awal et al., 2017; Waite et al., 2020; Ahearne et al., 2021), and descriptions from newly discovered myxobacteria often include predation data (Figure 2; Fudou et al., 2002; Sanford et al., 2002; Iizuka et al., 2003a,b, 2013; Reichenbach et al., 2006; Garcia et al., 2009, 2014, 2016; Mohr et al., 2012, 2018a,b; Yamamoto et al., 2014; Sood et al., 2015; Awal et al., 2016, 2017; Moradi et al., 2017; Garcia and Muller, 2018; Chambers et al., 2020; Livingstone et al., 2020; Wang et al., 2021a; Zhou et al., 2021). Myxobacteria are often split into two groups based on presumed nutritional preferences or needs, predatory myxobacteria that acquire nutrients from prey lysate and cellulolytic myxobacteria (Shimkets et al., 2006; Mohr, 2018). Representatives of cellulolytic myxobacteria are often described as non-predatory and include members of the genera *Sorangium* and *Byssovorax* (Korp et al., 2016; Perez et al., 2016; Petters et al., 2021). However, *Byssovorax cruenta* and seven recently discovered *Sorangium* spp. capably lyse Gram-negative bacteria and fungi (Reichenbach et al., 2006; Mohr et al., 2018b). These examples and previously mentioned inconsistencies between predatory capacity and phylogeny suggest that a binary grouping of myxobacteria as either predatory or cellulolytic is inaccurate. Instead, cellulolytic myxobacteria appear to support specialization related to nutrient acquisition, similar to observations of predatory specialization.

Methodological concerns over predation assay media types, specifically assays performed on solid agar versus liquid media, have introduced challenges to fundamental aspects of predation by myxobacteria. Comparing *M. xanthus* growth in liquid medium including either casein or hydrolyzed casein, Rosenberg et al. (1977) observed an increased growth rate in casein-supplemented media. The proportional association of secreted protease and cell number provided evidence of density-dependent growth and cooperativity during feeding. However, motility features of surface-dwelling myxobacteria do not enable swimming in aqueous conditions. Marshall and Whitworth suggest that this observed cooperative feeding may be an artifact of unnatural conditions of liquid cultures and instead propose myxobacterial predation to be an additive process that involves proportionate joint action of individuals (Marshall and Whitworth, 2019). Single-cell predation (Zhang et al., 2020), constitutive secretion of lytic enzymes (Livingstone et al., 2018a), and contact-dependent killing (Seef et al., 2021) all support myxobacterial predation being additive and not cooperative. Although predation assays on solid media better reflect soils and sediments, similar results between predation assays from aqueous and solid media have been reported. Myxobacterial selection of mucoid prey phenotypes has been reported separately from studies using either aqueous or solid media (Nair et al., 2019; Akbar and Stevens, 2021; Nair and Velicer, 2021). Interestingly, contact-dependent killing of *E. coli* by *M. xanthus* was previously reported for aqueous environments (Pan et al., 2013), and a type IV pilus similar to the Tad-like Kil proteins discovered from agar-based experiments was implicated (Seef et al., 2021). Overall, the necessity of environmentally-relevant solid media use in myxobacteria predation assays remains cogent but unsupported.

## Discrepancies between sequenced myxobacteria and environmental distribution

Previously classified as an order in the class *Deltaproteobacteria*, myxobacteria are now reassigned to the newly proposed phylum *Myxococcota* (Shimkets and Woese, 1992; Waite et al., 2020) which currently includes 2 classes, 4 orders, 7 families, and 31 genera. The majority of sequenced myxobacteria are members of the genera *Myxococcus* and *Corallococcus*. However, these genera are rarely or minimally present in environmental metagenomic data. Utilizing 10,000 samples from the Earth Microbiome Project (Thompson et al., 2017), Wang et al. (2021b) found myxobacteria are among the most diverse and globally ubiquitous bacteria on Earth. This study also observed ≥5 myxobacterial operational taxonomic units (OTUs) in every analyzed plant rhizosphere sample, and 1–5 myxobacterial OTUs in 95% of non-saline sediment and soil samples. Importantly, the vast majority of myxobacterial OTUs were unclassified at the genus level, and genera with >2 sequenced type strains each accounted for <2% of detected myxobacteria. Genera present in >2% of samples have only 5 sequenced representative type strains including *Haliangium ochraceum* DSM 14365^T^ (NC_013440.1), *Chondromyces apiculatus* DSM436^T^ (NZ_ASRX00000000.1), *Chondromyces crocatus* DSM14606^T^ (NZ_CP012159.1), *Labilithrix luteola* DSM27648^T^ (NZ_CP012333.1), and *Sandaracinus amylolyticus* DSM53668^T^ (NZ_CP011125.1). Similarly low abundances of myxobacteria from frequently sequenced genera have been reported from several biogeographical studies (Brinkhoff et al., 2012; Li et al., 2012; Zhou et al., 2014, 2020; Mohr et al., 2016; Wang W. et al., 2020; Petters et al., 2021). The discrepancy between genera of frequently sequenced myxobacteria and the geographic distribution of myxobacteria can be attributed to various challenges such as reliance on established isolation methods and underdeveloped cultivation techniques for lesser-studied genera. Mohr et al. (2017) discuss *Corallococcus*-specific myxospore recalcitrance to DNA-extraction which may also contribute to discrepancies between environmentally observed and cultivated myxobacteria. Notably, Wang et al. (2021b) did not detect any *Corallococcus* in the previously discussed Earth Microbiome Project study. Frequently sequenced myxobacteria are also the most often utilized in predation studies. Currently, there is little to no data for the predatory performance of abundantly distributed genera. In the Wang et al. (2021b) study, the genus *Labilithrix* was represented in ~3% of environmental samples compared to *Myxococcus* in 0.07% of samples. The original description of the lone type strain *L. luteola* DSM27648^T^ specifies an inability to lyse Gram-negative (*E. coli*) and Gram-positive (*Micrococcus luteus*) bacteria as well as *Saccharomyces cerevisiae* (Yamamoto et al., 2014), yet comparative genomic analysis of *Myxococcota* by Waite et al. included *L. luteola* among the myxobacteria predicted to be capable of pack-hunting (Waite et al., 2020). The discrepancy between myxobacteria typically included in predation studies and environmentally abundant myxobacteria limits understanding of the myxobacterial contribution to microbial community structure and nutrient cycling in Nature.

## Environmental factors that influence predation

Abiotic factors such as pH and certain alkaline earth metals influence myxobacteria populations in soils and compost manure (Zhang et al., 2013; Wang C. et al., 2020; Zhou et al., 2020; Dai et al., 2021). Increased actinorhodin production by *Streptomyces coelicolor* during co-culture conditions with *M. xanthus* has been associated with competitive acquisition of iron in soils (Perez et al., 2011; Lee et al., 2020), and copper detoxification has been observed to improve *M. xanthus* predation of *Sinorhizobium meliloti* (Contreras-Moreno et al., 2020). These observations suggest that environmental metal concentrations influence predation and myxobacteria presence in soils. Nonetheless, the direct effect of abiotic stressors on predatory performance with myxobacteria remains unclear. Microbial community diversity is the most consistently observed environmental factor influencing myxobacteria abundance and diversity. Bacteria in ecological studies from numerous orders have been positively correlated to interact with myxobacteria including *Anaerolineales*, *Burkholderiales*, *Cellvibrionales*, *Chitinophagales*, *Cytophagales*, *Flavobacteriales*, *Hyphomicrobiales, Ktedonobacterales*, *Pseudomonadales*, *Rhodospirillales*, and *Sphingomonadales* (Wang C. et al., 2020; Wang W. et al., 2020; Zhou et al., 2020; Dai et al., 2021). Often attributed to predatory preference of myxobacteria, connections between population-based ecological studies and predatory performance of myxobacteria remain unclear, and few environmental studies have provided genus-level correlations between myxobacteria and potential prey. Dai et al. observed a positive correlation between myxobacteria and various orders of Gram-negative bacteria and a negative correlation between myxobacteria and Gram-positive *Micrococcales* (Dai et al., 2021). Zhang and Lueders directly observed preferential myxobacterial predation of Gram-negative prey by introducing ^13^C-labeled *Pseudomonas putida* and *Arthrobacter globiformis* into an agricultural soil model system (Zhang and Lueders, 2017). However, *Haliangium* spp. were also capable of predating Gram-positive *A. globiformis*. Altogether, these environmental studies reinforce variation in predatory performance of myxobacteria by prey type originally observed from predator–prey assays (Morgan et al., 2010).

## Discussion

Continued multidisciplinary investigation of myxobacteria will help reveal how bacterial predators influence microbial community structure, enable continued discovery of therapeutic specialized metabolites, and inform soil health studies to improve agricultural outcomes. Determining roles of specialized metabolites during predation could improve prioritization of myxobacteria targeted for therapeutic discovery. Investigating the influence of predatory specialization on BGC evolution may inform synthetic biology approaches to refactor and utilize myxobacterial BGCs. Further study of pan-genome plasticity and any association with predation could reveal genetic determinants for predatory performance and prey range. Better understanding of predatory performance of lesser-studied myxobacteria abundant in soil, sediment, and plant rhizosphere may benefit application of myxobacteria as biocontrol agents. Ultimately, the potential advancement of any discussed concept or challenge provides compelling support for continued investigation of myxobacteria as generalist predators.

## Author contributions

KEP, SA, and DCS wrote and edited the manuscript. All authors contributed to the article and approved the submitted version.

## Funding

This work was supported by the National Institute of Allergy and Infectious Diseases (R15AI137996) and the National Institute of General Medical Sciences of the National Institutes of Health (P20GM130460).

## Conflict of interest

The authors declare that the research was conducted in the absence of any commercial or financial relationships that could be construed as a potential conflict of interest.

## Publisher’s note

All claims expressed in this article are solely those of the authors and do not necessarily represent those of their affiliated organizations, or those of the publisher, the editors and the reviewers. Any product that may be evaluated in this article, or claim that may be made by its manufacturer, is not guaranteed or endorsed by the publisher.
